# Impact of Social Determinants of Health and Demographics on Refill Requests by Medicare Patients Using a Conversational Artificial Intelligence Text Messaging Solution: Cross-Sectional Study

**DOI:** 10.2196/15771

**Published:** 2019-11-18

**Authors:** Rena Brar Prayaga, Ridhika Agrawal, Benjamin Nguyen, Erwin W Jeong, Harmony K Noble, Andrew Paster, Ram S Prayaga

**Affiliations:** 1 mPulse Mobile, Inc Encino, CA United States; 2 Grinnell College Grinnell, IA United States; 3 Medicare Medication Therapy Management & Medication Adherence programs Kaiser Permanente Southern California Downey, CA United States; 4 Regional Pharmacy Clinical Operations Kaiser Permanente Southern California Downey, CA United States

**Keywords:** text messaging, SMS, refill adherence, medication adherence, Medicare patients, conversational AI, social determinants of health, predictive modeling, machine learning, health disparities

## Abstract

**Background:**

Nonadherence among patients with chronic disease continues to be a significant concern, and the use of text message refill reminders has been effective in improving adherence. However, questions remain about how differences in patient characteristics and demographics might influence the likelihood of refill using this channel.

**Objective:**

The aim of this study was to evaluate the efficacy of an SMS-based refill reminder solution using conversational artificial intelligence (AI; an automated system that mimics human conversations) with a large Medicare patient population and to explore the association and impact of patient demographics (age, gender, race/ethnicity, language) and social determinants of health on successful engagement with the solution to improve refill adherence.

**Methods:**

The study targeted 99,217 patients with chronic disease, median age of 71 years, for medication refill using the mPulse Mobile interactive SMS text messaging solution from December 2016 to February 2019. All patients were partially adherent or nonadherent Medicare Part D members of Kaiser Permanente, Southern California, a large integrated health plan. Patients received SMS reminders in English or Spanish and used simple numeric or text responses to validate their identity, view their medication, and complete a refill request. The refill requests were processed by Kaiser Permanente pharmacists and support staff, and refills were picked up at the pharmacy or mailed to patients. Descriptive statistics and predictive analytics were used to examine the patient population and their refill behavior. Qualitative text analysis was used to evaluate quality of conversational AI.

**Results:**

Over the course of the study, 273,356 refill reminders requests were sent to 99,217 patients, resulting in 47,552 refill requests (17.40%). This was consistent with earlier pilot study findings. Of those who requested a refill, 54.81% (26,062/47,552) did so within 2 hours of the reminder. There was a strong inverse relationship (*r*10=−0.93) between social determinants of health and refill requests. Spanish speakers (5149/48,156, 10.69%) had significantly lower refill request rates compared with English speakers (42,389/225,060, 18.83%; X^2^_1_ [n=273,216]=1829.2; *P*<.001). There were also significantly different rates of refill requests by age band (X^2^_6_ [n=268,793]=1460.3; *P*<.001), with younger patients requesting refills at a higher rate. Finally, the vast majority (284,598/307,484, 92.23%) of patient responses were handled using conversational AI.

**Conclusions:**

Multiple factors impacted refill request rates, including a strong association between social determinants of health and refill rates. The findings suggest that higher refill requests are linked to language, race/ethnicity, age, and social determinants of health, and that English speakers, whites, those younger than 75 years, and those with lower social determinants of health barriers are significantly more likely to request a refill via SMS. A neural network–based predictive model with an accuracy level of 78% was used to identify patients who might benefit from additional outreach to narrow identified gaps based on demographic and socioeconomic factors.

## Introduction

### Background

Medication nonadherence is when patients are unable to follow prescribed treatment dose, time of day, and frequency [1]. There are a range of factors, including patient-related, physician-related, and health system barriers, that contribute to nonadherence [2]. Adherence has been shown to have a significant effect on treatment outcomes [3,4] and has a major impact in managing chronic conditions such as hypertension, cardiovascular disease, and diabetes. The Centers for Medicare and Medicaid Services (CMS) define an adherent patient as someone whose proportion of days covered is greater or equal to 80%. Put another way, patients are considered adherent when they refill often enough to cover 80% or more of their medication plan as prescribed by their health care provider and as agreed to by the patient [5]. Dispensing or refill data is commonly used to compute adherence levels because of the validity, relative accessibility, and inexpensiveness of such data [6].

The World Health Organization estimates a medication nonadherence rate of 50% for patients with 1 or more chronic conditions [2,7]. This staggering proportion of nonadherence is estimated to annually cost between 100 to 290 billion dollars in the United States [8]. Moreover, nonadherence is estimated to cause approximately 125,000 deaths and at least 10% of hospitalizations every year [9,10].

### Medicare Patients and Adherence

Individuals suffering from multiple chronic conditions and taking multiple medications are more likely to be nonadherent [11]. Patients eligible for Medicare, who are individuals older than 65 years and/or who have a disability, fit this description of patients at greater risk of nonadherence. Older patients and those with disabilities have more chronic conditions and are usually on multiple medications. Of 586 Medicare recipients offered medication therapy management, 575 (98.1%) completed a survey that asked questions relating to adherence. Among those who responded, 406 (69.2%) reported that they took their medication regularly and as prescribed. Of the remaining 169 (30%), 123 identified forgetfulness as an issue, 18 (11%) mentioned side effects, and 17 (10%) said the medication was not needed. Lower adherence rates were associated with difficulty paying for medication. Finally, subsidy recipients and non-English speakers were significantly less likely to be counseled about drug side effects [12].

### Use of Mobile Technology for Adherence

In a 2018 press release, the CMS committed to supporting modern and virtual methods of health care [13]. Furthermore, in a multinational survey conducted in 2018, 77% of respondents said the ability to request prescription refills via text message would increase their likelihood of choosing a health care provider. This percent is a 10-point increase over a 3-year span [14]. The Deloitte Center for Health Solutions conducted a nationally representative survey in which approximately a third of individuals indicated interest in receiving text messages for nutrition, exercise, sleep, and stress management [15]. These trends represent a changing societal landscape, and the health care field is poised to address this identified need. A recent interactive mobile solution for appointment reminders within the Department of Veterans Affairs (VEText) has been used to send SMS text message reminders to over 6 million veterans [16,17]. Text messages can also provide links to resources and reminders toward adopting healthier behaviors. Several meta-analyses corroborate the effectiveness of SMS for medication adherence [18-20]. An earlier study by the authors [21] measured the impact of SMS text reminders on refill rates of nonadherent and partially adherent Medicare patients with chronic disease. They found that text reminders increased refill rates by 14 percentage points compared with those who did not receive these reminders.

Important to the success of any intervention is its implementation, scalability, and sustainability [22]. Text messaging presents an effective, affordable, and scalable tool [21] that can use conversational AI to greatly impact health outcomes. More specifically, conversational AI (or conversational agents) can encourage health care consumers to engage with systems that imitate human conversations using text [23,24]. For example, a fully automated conversational AI system has been used to promote weight loss among overweight and obese diabetic patients [25]. As conversational agents can learn over time, interventions with thousands of users can be used to inform and improve the quality of the conversations, often within days or weeks. However, there is limited research currently available on the use of conversational AI within SMS (and not app-based) messaging for refill adherence.

### Social Determinants of Health

The World Health Organization has defined social determinants of health (SDOH) as the conditions or circumstances in which people are born, grow, live, work, and age [26]. The most commonly identified SDOH in the United States are housing, income, food, transportation, education, race/ethnicity, and unemployment [27,28]. Despite notable improvements in overall health over the past few decades, inequalities of SDOH contribute to persistent disparities in life expectancy and health outcomes [29,30].

Social determinants have also been linked to nonadherence, and a recent study using data from the National Health Interview Survey found that half of the adults with diabetes perceived financial stress, while one-fifth reported financial insecurity and food insecurity [30]. Since SDOH are not always recognized, they might be overlooked by a clinician in a medical setting. In a study of patients with chronic disease, two-thirds of those who reported not taking medications as prescribed due to cost never shared this with their physician [30,31]. The National Academy of Medicine has published a framework for educating health care professionals on the importance of SDOH [32], but there is still limited research studying the specific ways in which SDOH pathways interact to impact adherence, particularly in older populations.

### Objectives

A pilot study examining Medicare Part D patients over a period of 3 months supported the value of using SMS text message refill reminders to increase medication refill rates [21]. This study increases the sample size of those receiving SMS refill reminders to 99,217 Medicare recipients, includes both English and Spanish language text messages (the pilot used only English messages), and expands the duration to a 2-year follow-up period. This analysis focuses on a few different questions:

- First, were the results from the pilot study replicable with a much larger population using an enhanced version of the text messaging solution with improved conversational AI?
- Second, is there a relationship between SDOH and refill request rates, how large is this association, and do SDOH attenuate the association?
- Third, how do other variations in patient characteristics (age, gender, ethnicity, and language) moderate and predict likelihood of requesting a refill using a text message solution?

## Methods

### Participants

The SMS refill reminder program began as a 3-month pilot in December 2016 and was expanded to include multiple regions within a large integrated health system. The analysis covers a 2-year period from December 2016 to February 2019. It includes a population of 99,217 English- and Spanish-speaking patients (median age 71 years) targeted for medication refill by Kaiser Permanente, Southern California. The Kaiser Permanente, Southern California, Institutional Review Board determined that this program did not require review and was exempt.

All patients had Medicare Part D as their pharmacy benefits and had one or more chronic conditions (diabetes, hypertension, high cholesterol, and/or anticoagulation). Patients in this program refilled one or more of the following 4 classes of drugs: oral diabetes medications, blood pressure medications (renin-angiotensin system antagonists), statins, and direct oral anticoagulants (DOAC).

Targeted patients were shared by Kaiser Permanente, Southern California, in a weekly file, and they had varying levels of nonadherence. The total number of patients targeted for refill ranged from 1000 to 9000 patients per month. Note that these patients were not distinct each month because they could be on the list several times in a year. Patient records included first name, date of birth (DOB), gender, spoken language, address, race/ethnicity, mobile phone number, opt-in status, and refill drug(s). These fields, when available, were used for all analysis.

Tables 1 and 2 provide age and race/ethnicity breakdowns for this group.

**Table 1:** Age of text messaging group.

Age band (years)	Patients, n (%)	Reminders, n (%)
<60	6344 (6.39)	20,883 (7.64)
60-65	4502 (4.54)	13,806 (5.05)
65-70	29,600 (29.83)	79,349 (29.03)
70-75	27,201 (27.42)	75,902 (27.77)
75-80	15,039 (15.16)	42,221 (15.44)
80-85	7899 (7.96)	22,247 (8.14)
>85	5458 (5.50)	14,385 (5.26)
Unspecified	3174 (3.20)	4563 (1.67)
Total	99,217 (100)	273,356 (100)

**Table 2:** Race/ethnicity of text messaging group.

Race/ethnicity	Patients, n (%)	Reminders, n (%)
White	30,683 (30.93)	81,544 (29.83)
Hispanic/Latino	21,841 (22.01)	67,266 (24.60)
Black/African American	9124 (9.20)	28,365 (10.38)
Asian	8705 (8.77)	23,870 (8.73)
Other/mixed	1372 (1.38)	3812 (1.39)
Unspecified/unknown	27,492 (28.71)	68,499 (25.06)
Total	99,217 (100)	273,356 (100)

### Procedure

The intervention used the mPulse Mobile platform to deliver SMS text messages to patients. Patients in the text messaging group received a refill reminder dialogue that consisted of a series of messages written at a sixth-grade readability level.

Text message refill reminders were sent out on a weekly basis to patients who were due for a refill. The first message was a greeting, reminding patients that they were due for a refill. They were then prompted to validate their DOB (to ensure the person was the intended recipient of the reminder) by choosing from one of five options. If the patient validated their DOB successfully, they could then view their medication(s) due for refill and the last filling pharmacy. Patients did not get a second chance if they did not validate correctly. In those cases, a member from the pharmacy staff would reach out to discuss barriers to nonadherence and/or complete the refill by phone. As part of the refill workflow, patients could select whether to receive their medication by mail (this was added as an option in May 2018) or to pick up their medication(s) at a Kaiser Permanente outpatient pharmacy. They could also change their default pickup pharmacy. Pharmacy staff who were located at a central location were involved with managing the responses that came back from the patients. These patient responses were categorized as “Refill request,” “Barriers,” “Date of birth issue,” “Free text response,” “Side effects,” “Change Pharmacy,” and “Help.”

As in the pilot study [21], we used mPulse Mobile’s Engagement Console to support the pharmacy staff. This Web-based user interface allows users to quickly identify and prioritize subgroups of patients (as described above) for quicker follow-up.

**Figure 1:**
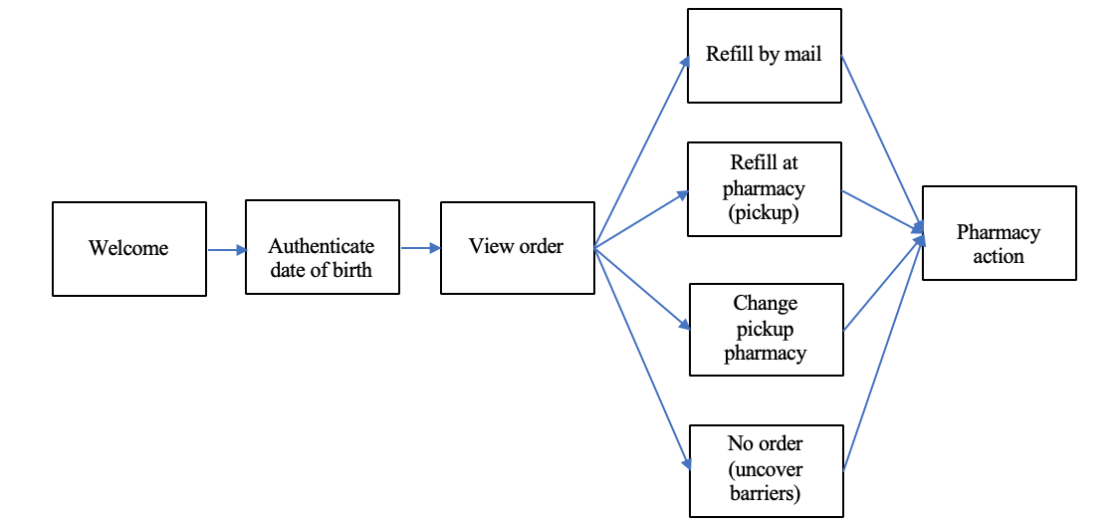
Overview of message flow within refill dialogue.

Figure 1 provides a view of the refill reminder message flow and the various steps or options within this dialogue. The initial step reminds the patients to refill the medication(s) and requests that they respond with the *structured options* “1” to continue or “2” to end. However, in some instances, patients respond with *unstructured responses* such as “I’ve already refilled” or “No thanks.”

A patient would receive a maximum of 3 messages if they did not respond (initial reminder, 2-hour reminder, and 24-hour reminder). The patient could opt out at any point during the messaging flow, and no subsequent messages would be sent.

Conversational AI was developed and used within the solution to also automatically process the following types of unstructured responses and patient requests:

- Patient wants to unsubscribe from texting program but does not reply “STOP” or 7867 (patients with older phones could use number keys to correspond to letters).
- Patient confirms intent to request a refill by using an unexpected phrase.
- Patient is experiencing side effects and might require medical attention.
- Patient wants to change pharmacy location where they wish to pick up the refill.
- Patient does not want to refill and provides a reason for not refilling.
- Patient requests help or wants additional information.
- Patient provides correct DOB instead of selecting a numeric option.
- Patient wants to switch language (English to Spanish or vice-versa).

For purposes of the qualitative analysis, each response was strictly coded as structured or unstructured. A response was considered *structured* if it exactly matched any of the following strings: *0, 1, 2, 3, 4, 5, 6, 7867,* and case insensitive versions of *AYUDA, HELP, MAIL, RESUB, STOP,* and *STOPALL*). All other responses were coded as unstructured.

Two coders classified each conversational AI rule using the description within the solution (eg, DOB validation and change order). There were a total of 75 conversational AI rules. These individual rules were then combined into 13 broader “rule type” categories: *initial reply, refill process, change pharmacy, DOB validation, barriers, payment, subscription, language change, member information, acknowledgment, feedback, help*, and *did not understand.* All rules belonged to the 13 rule types, and any ambiguity about rule type were resolved by discussion and agreement between the two coders.

An internally developed SDOH index was used to understand how unmet needs might impact patient refill behavior. When patient address was available, an SDOH index was computed. Multimedia Appendix 1 outlines the factors that were used to compute the SDOH index and to create low, medium, and high SDOH clusters.

A neural network multilayer perceptron (MLP) model was used to perform predictive analysis on factors that might impact refill requests.

## Results

The results of the scaled intervention are summarized in 3 parts: (1) a replication of the analysis performed in the pilot study; (2) results of subgroup exploratory analysis, including the use of an SDOH index and a predictive model; and (3) a qualitative analysis of the use and value of conversational AI and interactivity.

### Part 1: Analysis of Scaled Program

**Figure 2:**
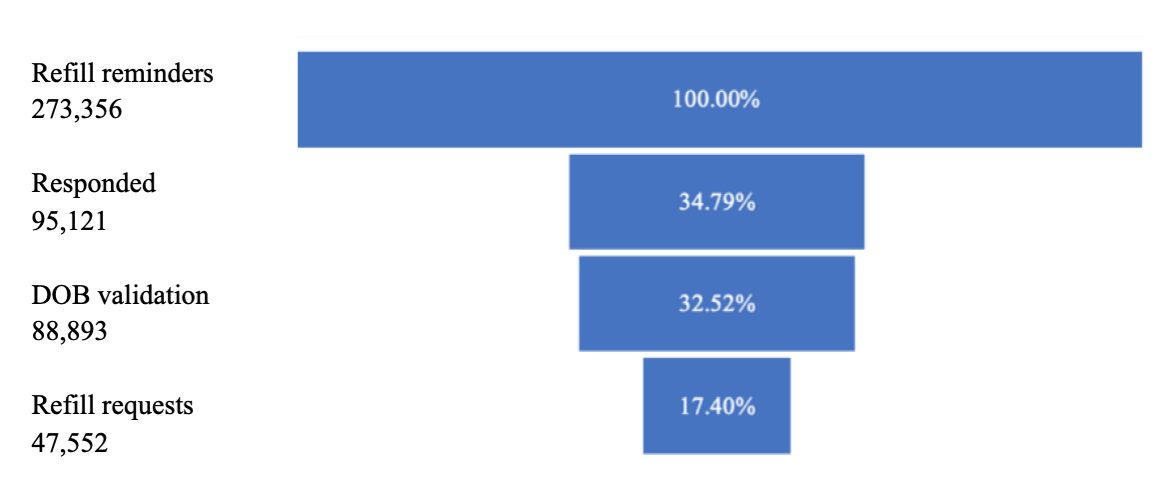
Conversion of refill reminder to refill request. DOB: date of birth.

A total of 273,356 SMS reminders were sent over a 2-year period to 99,217 Medicare Part D patients who had opted in to texting. In response, 17.40% (47,552/273,356) refills were requested. Figure 2 shows the conversion funnel from reminder to refill request.

**Figure 3:**
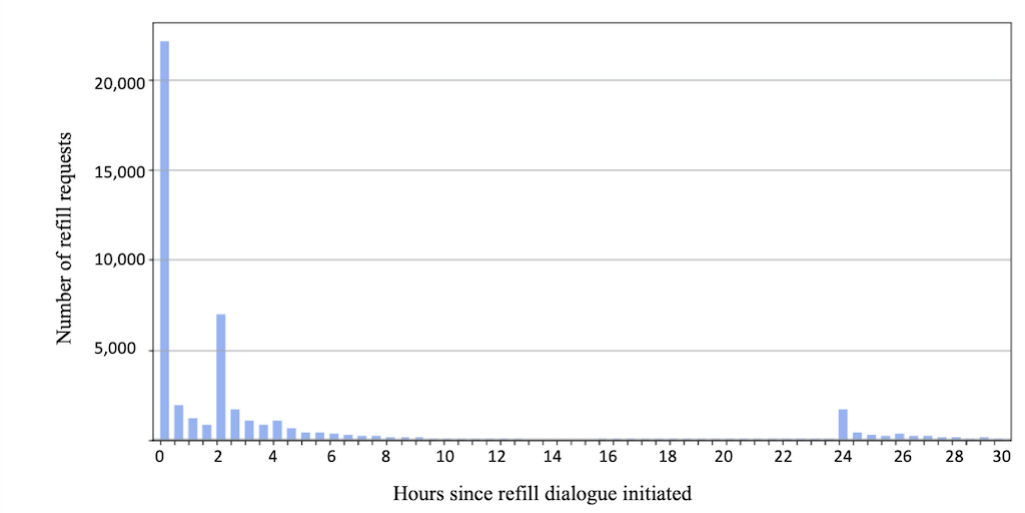
Refills requests by hour from initial reminder.

DOB validation was a necessary step to view the refill information, and this step resulted in a drop-off (DOB validation failures or did not attempt) of 6.55% (6288/95,121). Of those who requested a refill, 54.81% did so within 2 hours after receiving the initial reminder (N=26,062/47,552). As displayed in Figure 3, there are spikes in refill activity immediately after the initial message (“0”), after the 2-hour reminder (“2”), and the 24-hour reminder (“24”).

### Part 2: Exploratory Subgroup Analysis

We present the following subgroup analysis using 4 variables: SDOH, language, race/ethnicity, and age (gender did not have a significant moderating effect on refill rates).

#### Social Determinants of Health Analysis

**Figure 4:**
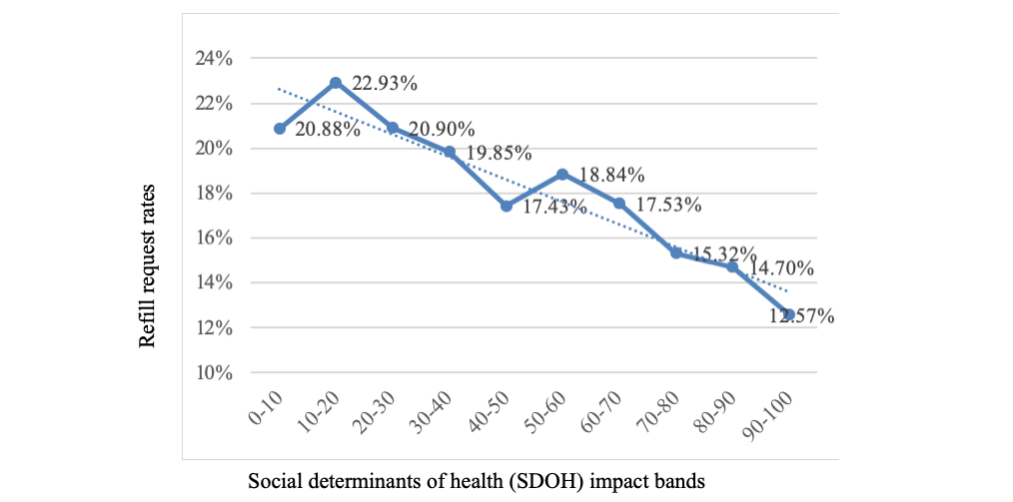
Refill rates versus social determinants of health bands.

Patients were grouped into 10 evenly spaced SDOH bands from 0 to 100. Refill requests were very highly inversely correlated with SDOH bands (*r*=−0.93), as shown in Figure 4.

To further understand the impact of SDOH on refill process, we grouped the SDOH bands further into 3 SDOH clusters (high, medium, and low) using k-means clustering as described further in Multimedia Appendix 1.

**Figure 5:**
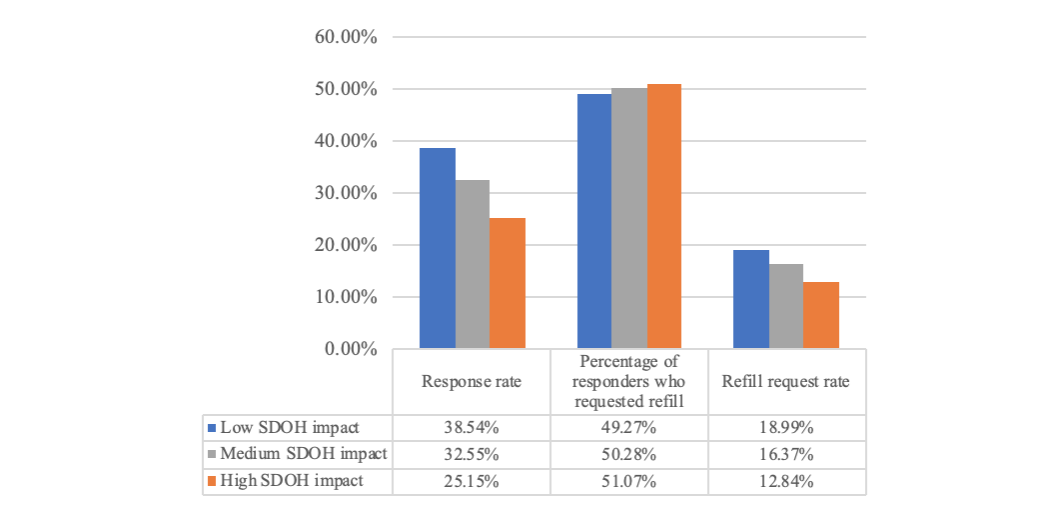
Social determinants of health impact on response rates and percentage of responders who request refill. SDOH: social determinants of health.

As can be seen in Table 3 and Figure 5, the negative correlation of refill request rates to SDOH index is driven primarily by the initial response rates (ie, after receiving the “Welcome” message in Figure 1, the patient confirms their intent to move forward in the dialogue). The difference in average SDOH between those who reply and do not reply was statistically significant (*t*_252,834_=−55.07; *P*<.001), but there was no impact of SDOH for refill requests after the patient engages with the initial text message (*t*_87,234_=1.71; *P*=.09). In other words, once a patient is willing to engage with the texting program, they request refills at the same rate regardless of SDOH levels.

**Table 3:** Refill request rate for text message group by social determinants of health level.

Social determinants of health	Refill dialogues, N	Percent who responded, n (%)	Percent of responders who requested refill, n (%)
Low (0-52.8)	124,423	47, 949 (38.54)	23,623 (49.27)
Medium (52.8-86.1)	94,489	30,755 (32.55)	15,464 (50.28)
High (86.1-100)	33,924	8532 (25.15)	4357 (51.07)

#### Spanish Versus English

Spanish-speaking patients had significantly lower refill request rates (5149/48,156, 10.69%) compared with English-speaking patients (42,389/225,060, 18.83%; X^2^_1_, [n=273,216]=1829.2; *P*<.001). As with SDOH impact, the initial response rates also vary by spoken language, where Spanish-speaking patients (8984/48,156, 18.66%) were far less likely to engage with text messaging than English-speaking patients (86,105/225,060, 38.26%; X^2^_1_, [n=225,060]=6716.9; *P*<.001).

Interestingly, a difference in refill request rates by language after the patient engaged with the reminder shows that Spanish-speaking patients request refills at a higher rate compared with English-speaking patients once they engage (5149/8984, 57.31% vs 42,386/86,105, 49.22%; X^2^_1_ [n=95,089]=212.5; *P*<.001).

We used a pointwise biserial correlation (point biserial correlation *r*_pb_=0.27; *P*<.001; N=252,696) to find that higher SDOH values are correlated with Spanish language preference. Spanish speakers (N=44,869) had a higher average SDOH of 71.78 as compared with English speakers’ (N=207,827) average of 55.65 (*t* =151.39; *P*<.001).

#### Age

Younger patients were significantly more likely to reply and request refills compared with older patients (*t*_83,415_=−43.30; *P*<.001). The older age group (75 years and older) responded at a rate of 29.84%, whereas patients younger than 45 years responded at a rate of 47.81%. There were also significantly different rates of refill requests by age band (X^2^_7_ [n=268,793]=1460.3; *P*<.001), with younger patients requesting refills at a higher rate, as shown in Table 4. We do see a spike in refill requests in the 85+ years group, and this suggests that caregivers or family members might be more actively assisting patients in this age band.

**Table 4:** Response and refill request rates by age.

Age band (years)	Refill dialogues, N	Responded, n	Date of birth validation, n	Refills requested, n	Request rate, %
<60	20,883	9388	8929	5277	25.27
60-65	13,806	5444	5061	2808	20.34
65-70	79,349	29,625	27,787	15,014	18.92
70-75	75,902	25,707	23,949	12,458	16.41
75-80	42,221	127,732	11,781	6103	14.45
80-85	22,247	6256	5780	3039	13.66
>85	14,385	4538	4242	2351	16.34
Unspecified	4563	1431	1364	501	10.98
Total	273,356	95,121	88,893	47,552	17.40

#### Does Race/Ethnicity Have an Influence on Engagement and Refill Request Rates?

**Figure 6:**
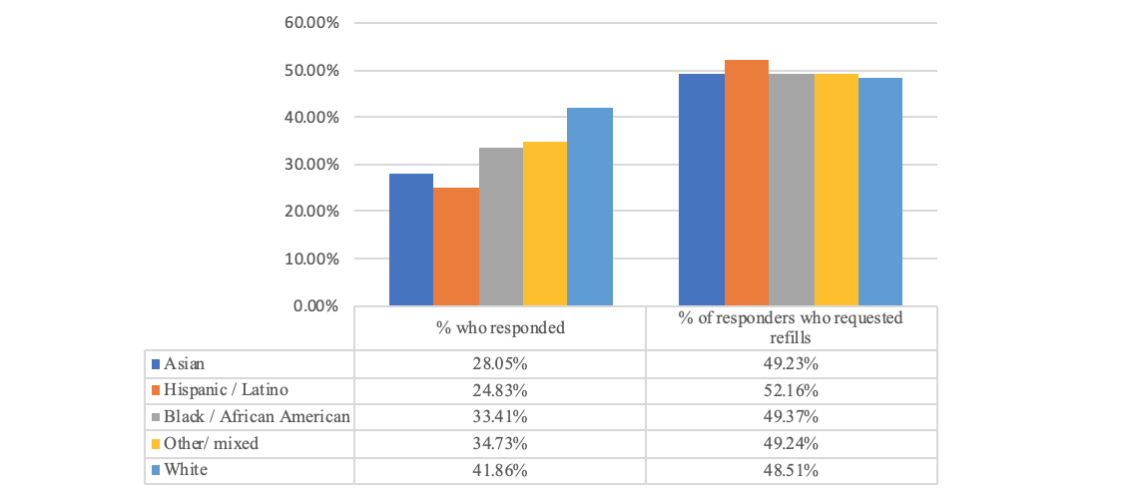
Impact of race/ethnicity on response rates and percentage of responders who request refill.

Patient race/ethnicity had a significant effect on initial response rates (X^2^_4_ [n=204,857]=5282.40; *P*<.001). Patients who identified as white responded at the highest rate (34,134/81,544, 41.86%), whereas Hispanic/Latino patients had the lowest response rate (16,700/67,266, 24.83%). Once someone did respond, race/ethnicity did not influence whether they refilled or not (X^2^_4_ [n=68,329]=2.37; *P*=.07), as reflected in Table 5 and Figure 6.

**Table 5:** Response and refill request rates by race/ethnicity.

Race/ethnicity	Refill dialogues, N	Responded, n	Date of birth validation, n	Refills requested, n	Request rate, %
Asian	23,870	6695	6233	3296	13.81
Hispanic/Latino	67,266	16,700	15,430	8711	12.95
Black/African American	28,365	9476	8751	4678	16.50
Other/mixed	3812	1324	1216	652	17.10
White	81,544	34,134	32,181	16,557	20.30

#### Predictive Model to Improve Refill Adherence

An in-depth analysis of the results revealed that the *reply rate* was the most important variable that we could influence to drive refill rates. After replying to the reminder, about half of the patients continue the conversation to validate their DOB and then request a refill of their medication(s), while the other half drop off and do not engage further.

As we were uncertain of the interaction of age, gender, SDOH, language, and race/ethnicity and how they moderate reply rates, we built a machine learning model using these factors as inputs to predict those least likely to reply. We trained a supervised model to predict reply likelihood based on known attributes. The model consisted of a neural network multilayer perceptron (MLP) with 24 input units, 10 hidden units, and a single output unit (to represent reply likelihood). The training set consisted of 70% of the available data, and model validation was done with the remaining 30%. The input vector consisted of features representing age, race/ethnicity, gender, SDOH, language, drug class, etc. We selected the most discriminative features to avoid overfitting and to reduce multicollinearity and redundancy in the feature space, and we used one-hot encoding to ensure uniform scale across features. Finally, we excluded rows with incomplete values. Each data row represented an instance of a unique series of events after a reminder was sent to a patient. This meant that if a member had been contacted 3 times to refill a drug and they only replied twice, the “reply” value would now be 0.66. This solved the problem of contradictory data and also converted the reply variable into a continuous variable representing reply likelihood.

#### Optimizing the Model to Predict Those Least Likely to Engage and Request Refill

**Figure 7:**
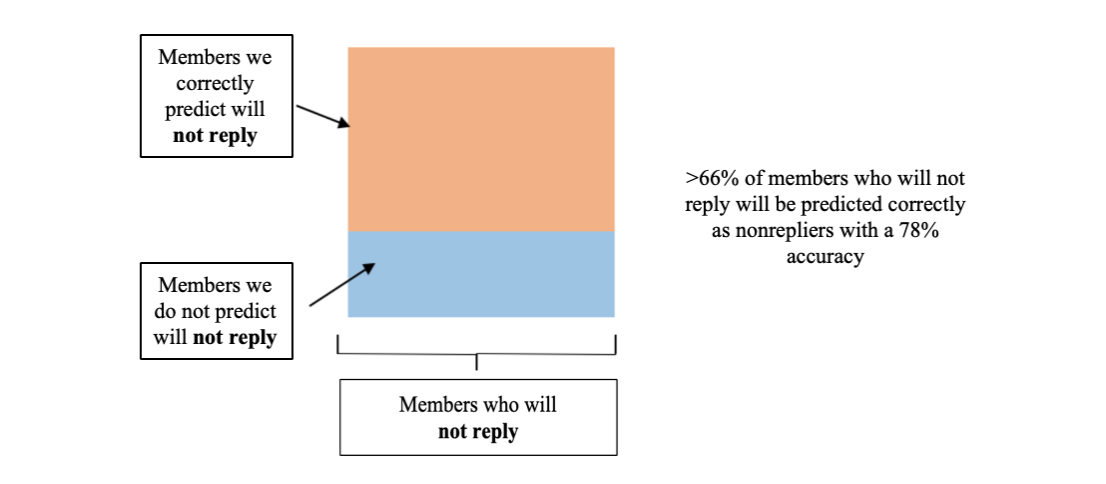
Minimizing the number of false positives.

To develop an approach to impact refill adherence, we wanted to first maximize the prediction accuracy for patients who were not likely to reply at all. We were less concerned about prediction accuracy for patients who were likely to engage and request refills. For the model to have value in an applied setting, we wanted to capture as many patients who were not likely to engage and might require additional support to request a refill. Figure 7 contains a visual representation of the confusion matrix, which highlights only those predictions that relate to nonreplying members.

To address these dual objectives (accurately predicting those who require outreach while maximizing the number of people who require outreach), we found a cut-off of 37% predicted likelihood of replying as a good threshold. This means that a patient whose predicted likelihood is less than 37% should fall into a category of requiring additional outreach. In summary, we can identify over 66% of those requiring outreach (because they will not reply) at a model accuracy of 78%.

### Part 3: Qualitative Analysis of the Usefulness of Conversational Artificial Intelligence

Our solution incorporated conversational AI (CAI) and natural language understanding (NLU) to provide a robust and successful interactive experience that ensures that the patient is able to request their refill as quickly and conveniently as possible.

We performed a qualitative analysis of all patient responses to evaluate whether the more complex capabilities of CAI and NLU were necessary and helpful for a better patient experience. As part of this analysis, we coded 307,484 responses as either structured or unstructured (as described in the Methods section).

Of the 307,484 responses that we received during the study period, only 7.77% (n=23,886) were not understood by the CAI. Table 6 provides a breakdown of structured and unstructured responses as well as the steps in the refill reminder dialogue where they typically occurred. All unstructured responses shown in Table 6 were understood by the CAI engine, which triggered the appropriate replies.

There were several instances when asked to provide structured response (eg, text 1 to view the medication), a member replies with additional information (eg, “1 – I only took this medication for 1 day and it caused great muscle pain. In the meantime, my cholesterol levels are now below 200”). Similarly, when asked to validate DOB by choosing from 1 of the 3 options, members will choose a number but also input the DOB as in “1 - 1950-05-30.” The system was largely successful in recognizing responses and accurately categorizing them. In Table 7, we present additional examples where the CAI was successful.

Textbox 1 includes a few sample responses that we failed to understand but could have handled appropriately if the patient had responded when expected or provided more context. In summary, our results indicate that despite the overall high accuracy (over 92%) of handling patient responses by the CAI, there are a number of instances (almost 8%) that were not handled at all, and in these cases, the patient was informed that the system was unable to understand their message.

**Table 6:** Counts of the type of responses that were handled by the conversational artificial intelligence (CAI).

Role of CAI	Structured responses	Unstructured responses	Total, n (%)
Supported by CAI	272,364	11,234	284,598 (92.23)
CAI could not handle	3001	20,885	23,886 (7.77)
Total	275,365 (89.55)	32,119 (10.45)	307,484 (100)

**Table 7:** Examples of unstructured patient messages when the conversational artificial intelligence successfully understood the message.

Sample patient responses	Response category
“1-I only took this medication for 1 day and it caused great muscle pain. In the meantime my cholesterol levels are now below 200”	Side effect barrier
“1/2 tablet twice or thrice weekly. Changed on 6/25/18 due to recurring muscle pain and Dr XXXX concurred”	Side effect barrier
“Both kinds of pain. I can’t walk very far if i take too many so I’ve cut it to 1/4th and then when it geta to bad i stop it for a few days.”	Side effect barrier
“Caused pain on calvrs so bad i could not walk”	Side effect barrier
“Doctor took me off medication because of too much muscle pain”	Side effect barrier
“I cant take a Statin It gives me terrible muscle pain and I cant sleep !!!! No.”	Side effect barrier
“I have reported to my previous primary and his medical assistant...that the dosage prescribed gave me leg cramps and pain...I agreed to take half a pi”	Side effect barrier
“Do not have the money right now to fill it will refill it a when I have the funds”	Cost barrier
“Don’t have money right now”	Cost barrier
“Don’t have the extra money till the first.”	Cost barrier
“Don’t have the money yet to refill but plan on refilling soon”	Cost barrier
“Filling px on the military base at no cost”	Cost barrier
“1 (one)”	“1”
“1 (sent with Invisible Ink)”	“1”
“1 proceed to refill”	“1”
“1 refill”	“1”
“1-”	“1”

**Sample of unstructured patient messages when the conversational AI responded to patients that it did not understand their message.**

Sample patient responses

“0 - This has already been ordered last weekend!”

“2 cuando lo recojo?”

“2 my body can’t tolerate this drug”

“A refill is not needed until the end of march.”

“Already ordered online”

“Cancel rx change”

“Doctor has lowered dosage from prescribed amount. Doctor needs to update prescription.”

“I am in San Diego now”

“I am out of the country”

“I need talk to Sam body”

“I want to talk to my doctor first.”

“Random”

“Still have full bottle of 60. Will order later.”

“What medication??”

## Discussion

### Principal Findings

We found that the refill request rates reported in the pilot study with English speakers [21] could be replicated at scale. The results in this study confirm and replicate the pilot results and reveal that, even after significant expansion of the population (scaled to over 7 times the initial group size and with the addition of Spanish speakers), the solution was effective in moving patients through the text dialogue to quickly complete a refill request. Pilot refill request rates of 18.1% were closely mirrored (17.40%), or even higher when limited to requests by English speakers (18.83%). Expanding the capabilities to include Spanish speakers was an important feature that is not commonly available in other texting solutions.

On the basis of the results in the previous section, we found that patients are not equally likely to request a refill. The findings suggest that good refill adherence is linked to language, race/ethnicity, age, and SDOH and that English speakers, white patients, those younger than 75 years, and those with lower SDOH barriers have significantly higher odds of requesting a refill via SMS. We studied these associations and developed a tool and approach that can be used for future outreach to narrow identified gaps based on demographic and socioeconomic factors and to increase overall refill adherence.

Finally, we wanted to evaluate the impact of the conversational AI engine, which is not typically found in other health care texting solutions. The results indicate that patients needed conversational AI as they traversed the refill reminder dialogue, and as health care consumers become more comfortable and familiar with artificial intelligence–based agents and chatbots, this expectation will only increase.

To our knowledge, no other published study (other than the study by Brar Prayaga et al [21]) has reported a refill adherence solution with results at scale [20]. The closest comparable solution with a large volume of patients is the VEText system for appointment reminders offered by the Department of Veterans Affairs, and we look forward to an in-depth study of that solution.

### Understanding Who Engages and Requests a Medication Refill

As our results indicate, we have identified several associations between factors that impact a patient’s likelihood to reply to the reminder and continue on to request a refill via SMS text messaging. These include language, SDOH, age, and race/ethnicity. For example, Spanish speakers were much less likely to engage with the reminder at all and, therefore, requested refills at a significantly lower rate of 10.69%, compared with English speakers at 18.83%. Note that for purposes of this text message solution, we messaged all patients in English (including those with a preferred language of Tagalog, Mandarin, etc in their health record) unless they had a preferred language of Spanish. Patients who identified as Hispanic/Latino had the lowest refill request rates of 12.95%, followed by Asian (13.81%), black (16.50%), and white (20.30%). Interestingly, neighborhood-based SDOH levels were highly correlated with patient language (English/Spanish), and patients with high SDOH barriers had significantly lower refill request rates of 12.84%, compared with medium SDOH (16.37%) and low SDOH levels (18.99%), as shown in Figure 5. Finally, younger patients were significantly more likely to reply and request refills compared with older patients. We believe these associations and effects should be addressed and have developed a predictive tool to help improve overall refill adherence rates for Medicare D patients.

### Using a Predictive Model to Assign Resources

A key goal is to increase the initial reply rate, and we were most interested in uncovering the population (roughly two-thirds) who were *unlikely to engage at all.* We found that the predictive model is able to accurately (>78%) pinpoint a high percentage (>66%) of patients who will not engage with an SMS text reminder to request a refill. This can be used as a valuable tool by a health provider or pharmacy to proactively communicate with populations who are least likely to complete a refill request. While current outreach methods to encourage adherence, such as phone calls by pharmacy staff or automated interactive voice response calls, are typically more costly and time consuming [21,33], a targeted approach using the predictive model could optimize limited staff resources.

### Addressing Barriers to Improve Refill Adherence

Another recommendation is to reduce the *initial barriers* or unwillingness to engage with the program. For example, lack of familiarity with texting or mistrust of the channel, especially among older patients, could be addressed with more tailored versions of the initial reminder that alleviate possible unease with using SMS text messaging to complete a refill transaction.

Similarly, health plans and providers could provide supplemental resources (such as an informational video to explain the process for requesting a refill via text message and to address any specific concerns of Spanish speakers), and the text dialogue could link to these resources. It might also be beneficial to add multilanguage support to expand beyond English and Spanish. In addition, as cost can be a barrier to refill for patients, especially for those with high SDOH levels, the text message dialogue could remind patients that, for instance, mail order refills are incentivized (eg, “did you know that if you order by mail you can get a 3-month supply of medication for the cost of a 2-month supply?”).

We also found that for a large percentage of refill reminders (15.75%, n=14,005), patients start the refill process (ie, view medication and validate their DOB) and then share a barrier or other concern that causes them to drop out of the process. There were an additional 10,192 instances where patients shared a reason for not refilling in other parts of the conversational flow. These barriers include a wide range of topics such as cost, side effects, already refilled, still have sufficient medication, do not want to refill, and taking differently than prescribed. We are sharing this response data with Kaiser Permanente, Southern California, and they are continuing to find ways to address these issues and follow-up with patients as required.

### The Role of Conversational Artificial Intelligence

Conversational AI was helpful in moving patients through the refill dialogue using mostly structured inputs almost 90% of the time. Although complex conversational AI was not essential for driving outcomes (validating DOB, completing refill, etc), it played an important role in keeping the conversation going when patients engaged using unprompted or unstructured messages (“Caused pain on calvrs so bad I could not walk [sic]”). In this example, it is recognizing the patient’s expressed concern as a *side effect*, thereby allowing the member to respond as they see fit to continue the conversation instead of being constrained by a rigid structure. This model of primarily relying on prompted responses and also understanding those cases where users want to go outside a closed response set allows the user to control the flow of the conversation in keeping with Grice’s Maxim of Manner [34]. Press releases relating to the recent launch of VEText [16] suggest a significant impact on appointment no-show rates at scale (there is no peer-reviewed study on the solution currently available, but the press release reported a no-show reduction from 13.7% to 11.7%, N not reported). The system requires users to only use prompted and structured alphanumeric responses, such as R2 and K3 [17]. While the details of the VEText solution are unclear, support for unstructured responses (estimating 10% based on our results) using conversational AI would likely improve user experience as well as overall outcomes. We believe this hybrid approach of supporting dual modes of interaction will support a fluid and frictionless conversation to enable task completion and allows a more empathetic exchange with the health care partner. Finally, while our accuracy rate of conversational AI was over 92%, we continue to explore ways to improve the system. A review of the literature using conversational AI reveals that the focus area is limited in scale and tends to be restricted to apps [24] and not SMS.

It is unclear why there is limited adoption of conversational AI within SMS text messaging as this is a channel with potential to reach all segments of the population. This study is unique in analyzing a text messaging and conversational AI solution at scale that allows elderly populations to easily and conveniently request their medication refills.

### Limitations

The findings of this study have to be seen in light of some limitations. The study was not a randomized controlled trial, and there is the possibility of selection bias. Due to regulations within the wireless communication industry and the Telephone Consumer Protection Act, we must have prior consent before messaging patients, and this constraint applies to any automated text messaging solution. As a result, the study targeted only those patients who had already opted into digital engagement. As the messaging program requires that patients have a mobile number with a texting plan, patients with only landline numbers were excluded. The nontext group received phone call outreach as part of the standard of care. However, we have already demonstrated the value and incremental benefit of SMS text reminders as compared with phone reminders [21], and this was not a focus of the study.

Using bivariate analyses can inflate the type 1 error, and this is a limitation. At the same time, we attempted to address this issue by using a neural network predictive model, which is a form of multivariate regression and can reduce the impact of multicollinearity. In addition, we did not address the impact of multiple reminders over time—that is, does a patient who requests a refill after receiving a reminder, and later becomes nonadherent again, continue this positive behavior upon receiving a future reminder?

Finally, the solution described in this study is a commercial system offered by mPulse Mobile with a licensing fee. We are unable to share financial benefits of using the system, such as operational efficiencies, health savings, and reimbursement revenue from improved adherence, as this information is proprietary and confidential. At the same time, we cannot disclose solution costs (such as messaging costs, license fees, and implementation costs). However, the overall financial gains from using the refill reminder solution were greater than system costs.

### Conclusions

Overall, this study indicates that there are sharp differences in likelihood to reply to a refill reminder and complete a refill request via SMS based on demographic and socioeconomic factors. We found a strong association between refill request rates and patient language, age, race/ethnicity, and SDOH levels, and these differences may contribute to health disparities and impact health outcomes in Medicare patients. Using a predictive and innovative model to target patients least likely to engage with the SMS solution and crafting a tailored mobile communication and conversational AI strategy could reduce these inequalities and improve refill adherence. We will continue to refine our solution and optimize our predictive model to validate our results and hope to be able to address disparities and drive even stronger outcomes. Finally, we believe that, to ensure the success of a text messaging solution and yield similar results, message tone and content, ease of use, level of tailoring, and quality of conversational AI are important considerations.

## Appendix

Multimedia Appendix 1 Calculating the Social Determinants of Health Index.

## Abbreviations

AI: artificial intelligence
CAI: conversational artificial intelligence
CMS: Centers for Medicare and Medicaid Services
DOB: date of birth
MLP: multilayer perceptron
NLU: natural language understanding
SDOH: social determinants of health
