# Supplementary material for: Impact of Social Determinants of Health and Demographics on Refill Requests by Medicare Patients Using a Conversational Artificial Intelligence Text Messaging Solution: Cross-Sectional Study
Source: JMIR Mhealth Uhealth. 2019 Nov 18;7(11):e15771. doi: 10.2196/15771 (PMC6887813; doi:10.2196/15771)
Supplement: Multimedia Appendix 1 [file mhealth_v7i11e15771_app1.pdf]

Appendix A

The SDOH index takes into account six categories identified by CDC and the Kaiser Family Foundation [25, 26]: Economic Stability, Social and Community Context, Education, Neighborhood and Built Environment, Health and Health Care and Food. This index provides a granular view of the population at the United States census tract level and can be used to highlight neighborhoods where there might be a higher incidence of unmet social needs and an increased likelihood of health inequities. The index has a range of 0 to 100 where 0 represents a low needs census tract and 100 represents a high needs area.

Indicator	***SDOH category***	***SDOH sub-category***	***Description***
Percent ethnic diversity	Community and Social Context	Social integration/discrimination	Percentage of people in a census tract who are any race other than Caucasian
Percent households SNAP benefits	Food Insecurity	Access to Food	Percentage of households in a census tract receiving SNAP benefits
Percent no car	Neighborhood and Built Environment	Transportation Access	Percentage of households in a census tract without a vehicle
Percent no high school	Education Attained	Higher education	Percentage of people in a census tract without a high school diploma
Percent no insurance	Health and Health Care	Health coverage	Percentage of people in a census tract without health insurance
Percent poor English	Education Attained	Literacy	Percentage of people in a census tract who speak English less than very well
Percent poverty	Economic Stability	Income	Percentage of people in a census tract living below the poverty line
Percent renters	Neighborhood and Physical Environment	Housing Instability	Percentage of living units in a census tract who rent their place of residence
Percent unemployment	Economic Stability	Employment	Percentage of people in a census tract who are unemployed

Table A: Factors used to compute SDOH index.

To create low, medium, and high SDOH index groups, k-means clustering (k=3) was performed on census tract variables/indicators that compromise the SDOH index, such as percent owning a car and percent without insurance. A histogram of counts of census tracts for each SDOH index value, grouped by k-means clusters showed two boundaries to divide groups. The resultant SDOH index value groups are as follows: the low SDOH group corresponds to SDOH index values between 0.00 and 52.80, the medium SDOH group is values 52.80 to 86.10, and high SDOH group is values 86.10 to 100.00. The yellow lines indicate where the low-medium (52.80) and medium-high (86.10) boundaries were drawn.

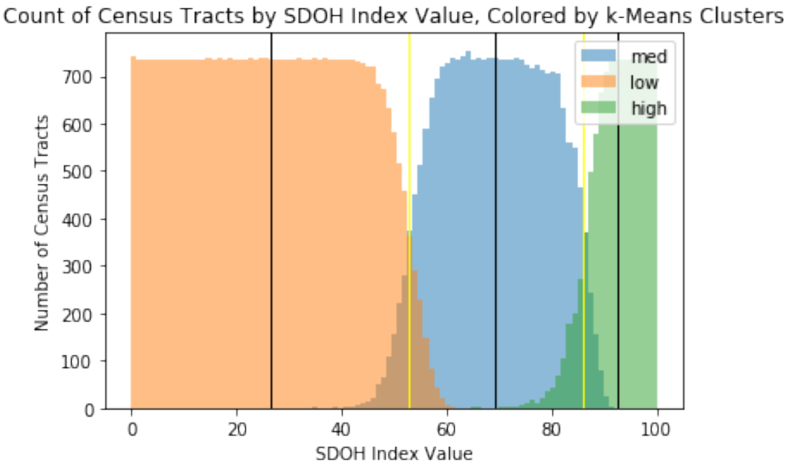

Figure A: Histogram of SDOH index value colored by groups created using k-means (k=3).
